# Medical Matters, &c., in Geneva

**Published:** 1845-11

**Authors:** 


*Medical Matters, &c. in Geneva.* From Prof. F. H. Hamilton's "Notes of an European Tour," published in the Buffalo Medical Journal, we extract some observations on Geneva in Switzerland.—At Geneva I have spent nearly a week, which has afforded me time to visit all places of special interest. My first business was to call upon Dr. H. C. Lombard, physician to the Civil and Military Hospital of Geneva, whom I found exceedingly attentive, and to whom I am indebted for much valuable information. Dr. L. is not, I think, over 35 years of age, yet he has already greatly distinguished himself as a writer and practitioner. His medical education was acquired mostly in Paris, but he spent sufficient time in England to render him familiar with English practice, and to obtain such a knowledge of the language as to enable him to speak and write it handsomely. Although he had made his morning rounds, he kindly offered to accompany me to the hospital, a fine stone building forming a spacious court and situated in the upper part of the city. The first thing which arrested my special attention was the prevalence of goitre, with which not only the patients but the nurses almost without exception were more or less affected. To the inquiry whether it was not more common with women than men, Dr. L. replied that he did not think it was with unmarried women, but that in those districts where goitre most prevailed, its developement was almost certain after child-birth, and that even at Genəva, English and other foreign ladies were exceedingly apt to become affected with goitre immediately succeeding parturition. At Geneva and in its immediate vicinity, the proportion affected with this disease is not so large, but Dr. L. assured me that in the "Valais Canton" about two thirds of the population were goitrous, the absence of the usual appendage being regarded as a deformity! In reference to its cause, a point so much in dispute, Dr. L. remarked that at Geneva, it was probably not true, as has been stated, that those who drank the lake water were less liable to the affection than those who drank water at certain springs in the city, and that in other parts of Switzerland I would find it prevailed mostly in deep valleys, and especially along those which extended north and south, and from which the direct rays of the sun were therefore mostly excluded. I also remarked to him what seemed to me to have some bearing upon the question, that the great mass of his hospital patients were scrofulous; almost every one, under whatever other malady they might be suffering, if the malady was chronic, had superadded also either enlargements of the glands of the neck, or chronic ophthalmia, or tumefaction of face or lips, or spinal distortion, or coxalgia, or enlargements of the knee, ankle, or of smaller joints. One, a girl about 17, I remember well, as presenting a most hideous picture, the very "tout ensemble" of scrofulous disfigurement; for in addition to many of the local affections already enumerated, her right eye was half protruded from its socket by an enormous irregular tumor, situated upon the antrum, discharging matter at several points, and the whole space between her chin and sternum was occupied by a large, nobby, ugly-looking goitre.

The conclusion to which I have arrived, then, as to its cause, is that it depends upon the same causes, only slightly modified, which usually develope scrofula, an opinion which I shall hold, with the right of change, until I have myself visited the goitrous districts, as I propose soon to do.

"First of all," says Dr. L., "the patient, if we would cure him, must be removed from the valley to the mountain, and then," adds Dr. L., "I consider iodine as much a specific as quinine is for intermittent fever, and quite as certain, provided the remedies are applied early." He exhibits iodine both internally and externally, having become of late somewhat cautious to avoid iodization, an event which is indicated by a general and rapid marasmus, hectic, and often speedy death. In the only instance in which Dr. L. had seen the thyroid gland removed, the patient died of tetanus; Dr. Bizot, the surgeon, however, remarked that he had removed safely encysted goitre, but would never attempt the removal of a simple thyroid hypertrophy. He also stated that he had found the "*huile de foi de morue*"—cod liver oil—a most excellent substitute for iodine in certain cases, and that he had also extended its use beneficially to cases of simple scrofula; the muriate of gold, also, he relied much upon in scrofula. The observations of these men I regard of unusual value, from the acknowledged accuracy and honesty of the gentlemen and their unequalled familiarity with the diseases in question. In the surgical wards of Dr. Bizot I saw nothing strikingly peculiar; the straight splint, in its simplest form, is here generally employed, and, indeed, as the Swiss surgeons are generally educated at Paris, their surgery differs but little from that of the Parisian hospitals.—*Bost. Med. and Surg. Jour.*

